# Health-related quality of life in primary care patients: a comparison between EQ-5D-5L utility score and EQ-visual analogue scale

**DOI:** 10.1186/s12955-023-02215-w

**Published:** 2024-01-03

**Authors:** Richard Huan Xu, Ruiqi Sun, Lidan Tian, Annie Wai-ling Cheung, Eliza Laiyi Wong

**Affiliations:** 1 Department of Rehabilitation Sciences, Hong Kong Polytechnic University, Hong Kong, China; 2 JC School of Public Health and Primary Care, The Chinese University of Hong Kong, Hong Kong, China

**Keywords:** Health-related quality of life, EQ-5D; EQ-VAS, Utility, Measurement properties, Primary care

## Abstract

**Objective:**

The EQ-VAS is an important component of the EQ-5D questionnaire. However, there is limited evidence comparing its performance to the EQ-5D utility score, which restricts its use in the population. This study aimed to EQ-5D-5L utility score and EQ-visual analogue scale (EQ-VAS) in primary care patients in Hong Kong (HK).

**Methods:**

Secondary data analysis was performed on the data collected from a cross-sectional survey to investigate patient engagement in HK. Participants were recruited through random sampling from a single general outpatient clinic. Trained investigators conducted face-to-face interviews with all eligible patients attending the clinic. Patients who were: 1) ≥ 18 years old, 2) have visited the clinic at least once in the last 6 months, 3) no cognitive problems, and 4) can speak and understand the local language. Pearson correlation was used to explore the association between EQ-5D utility and EQ-VAS score. Ordinary least squares regression and heteroscedastic Tobit regression models were adopted to analyze the EQ-VAS and EQ-5D utility data, respectively.

**Results:**

The analysis included data from 1,004 responses (response rate = 65%). Around 52.7% of participants were female, 25.9% completed tertiary or above education, and 75.1% living with chronic disease. The mean EQ-5D utility and EQ-VAS score were 0.92 (SD = 0.13) and 72.27 (SD = 14.69), respectively. A significant association was found between EQ-5D utility and EQ-VAS score, with coefficients ranging from 0.335 (participants who divorced) to 0.744 (participants living alone). Around 98.5% reported having no problems with 'Self-care', followed by 'Usual activities' (96.3%), 'Mobility' (91.5%) and 'Anxiety/depression' (79.9%). The correlation between EQ-VAS score and EQ-5D utility was positive for each dimension of the EQ-5D instrument (correlation coefficients ranged between 0.211 and 0.623). Age strongly influenced the magnitude and trajectory of EQ-VAS score and utility, as observed in the changes. The regression model showed that 'Mobility', 'Pain/discomfort', and 'Anxiety/depression' have considerable influence on EQ-VAS score.

**Conclusions:**

This study compared the EQ-5D utility score and EQ-VAS in HK primary care setting. Although heterogeneity existed, the EQ-VAS and utility score are significantly correlated and reliable for evaluating health-related quality of life in this population.

**Supplementary Information:**

The online version contains supplementary material available at 10.1186/s12955-023-02215-w.

## Introduction

 Patient-reported outcome measures (PROMs) are increasingly recognised as useful tools to help healthcare providers gain a comprehensive understanding of the ‘value’ of a healthcare system and to provide patients with an opportunity to express their preference for healthcare services [1, 2]. The EuroQol five-dimensional (EQ-5D) instrument, which was introduced by the EuroQol group in 2005, is one of the most famous and widely used PROMs worldwide [3]. Given its nature of generic attribute, EQ-5D has been recommended in recent years by an increasing number of health technology assessment (HTA) bodies worldwide, and thus, it has been widely used to estimate quality-adjusted life years in cost–utility analysis [1].

The EQ-5D questionnaire consists of two parts. The first part is a descriptive system that contains five dimensions; it can be used to estimate the utility index. The second part is the EuroQol visual analogue scale (EQ-VAS), which is a 0–100 scale where respondents indicate their overall health status. The descriptive system and EQ-VAS can separately derive utility and EQ-VAS score to reflect the respondents’ health status in different scales. Utility is frequently used to refer to the preference of a patient for a particular health outcome attribute [4]. EQ-VAS is a global assessment of health patients provided [5]. The EuroQol group recommends using both tools to estimate the health status of people and then report the results based on different profiles. However, the literature review indicates the most common approach to describe EQ-5D data is to report only the single number of utilities derived based on a local value set [6], which makes analysis considerably easy. Derret et al. found that 50% of EQ-5D studies report only the EQ-VAS result [7].

As a possible measure for economic evaluation, VAS was developed approximately 40 years ago and is currently underreported [4]. VAS can still contribute to health-related quality of life (HRQoL) assessment because of its high response rate, high completion level, and low cost [5, 8]. At present, EQ-5D is widely used in studies on clinical interventions, patient experiences, and cost-utility analyses [9–12]. Search through Dimensions indicated that more than 1,200 publications related to EQ-5D were found in 2018, however, only a few publications reported the association between utility and EQ-VAS in general population and patient groups. For example, a UK study indicated that EQ-VAS data yield different results compared with the utility index in patients using National Health Services and can measure a broader underlying construct of health [5]. Another Swedish study suggested that adolescents can value their own health state using the VAS and capture aspects that are important for them [13]. A study in Thailand also indicated that the EQ-VAS showed some better measurement properties than the EQ-5D utility score, but poorer than SF-6D utility score [14]. Removing EQ-VAS data from the analysis or reporting only a simple mean value of these data has apparently become a trend based on the increasing number of EQ-5D studies with such characteristics. To the best of our knowledge, no study in Hong Kong (HK) has yet discussed the roles of the utility index and EQ-VAS or their association with each other in HRQoL studies. This should not only be methodological but also practical for assessing people’s HRQoL.

In recent decades, the EQ-5D is an increasingly important and widely utilized tool in primary care settings. Its usage has been steadily rising as healthcare professionals recognize its immense value in assessing patients' HRQoL [15, 16]. By incorporating the EQ-5D into primary care practices, healthcare providers can gather valuable data on patients' overall well-being and make more informed treatment decisions. This standardized measurement allows for consistent evaluation and comparison of patients' health status across different primary healthcare settings and populations. As healthcare providers strive to deliver patient-centred care and improve health outcomes, the EQ-5D serves as a valuable instrument in understanding and addressing the holistic needs of patients. Several systematic reviews confirmed that the VAS is a valuable tool to support economic evaluations, and more research on the role and consequences of using the VAS is warranted, especially, in Asian population [17, 18]. Therefore, this study aims to present and compare the utility score of the five-level EQ-5D (EQ-5D-5L) and EQ-VAS in primary care patients; and also explores their association, adjusted for sociodemographic characteristics, based on data from the general outpatient clinics (GOPCs) in Hong Kong (HK).

## Materials and methods

### Setting

The GOPC is established by the HK Hospital Authority to provide comprehensive primary care services to residents of all ages and across all districts. It offers a wide range of services, such as general medicine, geriatrics, routine check-ups, preventive screenings, and treatment for acute or chronic conditions. The clinic aims to meet the diverse healthcare needs of the community. Moreover, if a patient requires further evaluation or specialized treatment, the clinic's healthcare professionals can make timely and appropriate referrals to ensure that patients receive the possible care. To improve the representativeness of our sample, we compared our sample to the general population of Hong Kong to ensure the validity of our findings.

In this study, we have collected data from *Fanling Family Medical Center* in North district of HK, which providing serves for more than 0.3 million residents. The Fanling Family Medical Centre provides the biggest volume of services in North District, 2^nd^ biggest volume of services in New Territory East and 12^th^ for the whole HK. Like other GOPCs operated by the Hospital Authority, the *Fanling Medical Centre* primarily serves two categories of patients: those with chronic diseases and stable conditions, and those with episodic diseases and relatively mild symptoms. In addition, it offers nursing services such as drug injection and wound dressing. Moreover, GOPCs provide health risk assessments and follow-up care for patients with conditions like diabetes mellitus or hypertension through multi-disciplinary teams. They also offer targeted treatment services, including continence care and medication management, for high-risk chronic patients with the assistance of nurses and allied health professionals such as physiotherapists, occupational therapists, and pharmacists.

### Study design and population

The data used in this study obtained from a cross-sectional study that conducted between May and August to evaluate the validity of a newly developed questionnaire for testing patient engagement in healthcare services. A team of well-trained investigators conducted face-to-face interviews to collect data in the target outpatient clinic. The inclusion criteria for participants were as follows: 1) ≥ 18 years old, 2) have visited the clinic at least once in the last 6 months, 3) no cognitive problems, and 4) can speak and understand the local language (i.e., Cantonese). The original study was divided into two stages with different purposes. The first stage aimed to assess the psychometric properties of a newly developed instrument and collected a sample of 318 participants. The second stage of the study aimed to assess the level of patient engagement in using primary care services and collected a sample of 686 participants. Finally, data from 1,004 participants were used for the analysis in this study. The details of the original study were reported in these papers [19, 20]. We compared our sample with the general population in Hong Kong and found that, except for a slightly higher proportion of older respondents, there was no significant difference in terms of sex, educational level, governmental allowance, and marital status (Appendix, Table A1). However, this proportion is consistent with the characteristics of patients who avail of GOPC services in HK.

**Table 1:** The demographics of respondents and the correlation between EQ-5D utility and EQ-VAS score

	**N**	**%**	**EQ-VAS (SD)**	***p*** **-value**	**Utility (SD)**	***p*** **-value**	**Correlation coefficient**
**Age (Mean, SD)**	50.91 (14.45)		72.27 (14.69)		0.92 (0.13)		
**Sex**							
Male	475	47.3	71.88 (14.19)	0.514	0.93(0.12)	** < 0.001**	0.491***
Female	529	52.7	72.61 (15.12)		0.91(0.13)		0.571***
**Educational attainment**							
No education/Primary	151	15.0	69.90 (15.23)	0.115	0.90(0.15)	**0.042**	0.498***
Middle school	259	25.8	71.25 (14.61)		0.91(0.15)		0.628***
Senior school	344	33.3	73.14 (14.58)		0.92(0.12)		0.498***
Tertiary or above	260	25.9	73.53 (14.43)		0.94(0.09)		0.487***
**Living status**							
Live alone	71	7.1	71.41 (16.44)	0.327	0.89(0.16)	0.347	0.744***
Live with family	927	92.7	72.32 (14.57)		0.92(0.12)		0.508***
Others	3	0.3	NA		NA		NA
**Working status**							
Retired	232	23.1	70.99 (15.33)	0.184	0.92(0.14)	0.079	0.550***
Unemployment	36	3.6	68.61 (17.71)		0.88(0.17)		0.705***
Full-time student	39	3.9	73.10 (15.94)		0.93(0.11)		0.519***
Housewife	189	18.8	71.34 (16.34)		0.90(0.16)		0.624***
Full-time employment	489	48.7	73.29 (13.40)		0.93(0.10)		0.438***
Part-time employment	19	1.9	75.79 (9.76)		0.90(0.13)		0.368
**Marriage**							
Single	233	23.2	70.88 (15.51)	0.421	0.92(0.11)	0.224	0.566***
Married	712	70.9	72.77 (14.49)		0.92(0.13)		0.531***
Divorced	34	3.4	70.44 (14.94)		0.90(0.12)		0.335*
Widow	25	2.5	73.20 (11.35)		0.89(0.13)		0.643***
**Government allowance**							
Yes	93	9.3	68.12 (19.15)	0.071	0.85(0.18)	**0.002**	0.509***
No	911	90.7	72.69 (14.10)		0.93(0.12)		0.609***
**Chronic condition**							
Yes	754	75.1	71.55 (14.73)	**0.002**	0.92(0.14)	0.994	0.534***
No	250	24.9	74.44 (14.35)		0.93(0.10)		0.532***
**Caregiver**							
Yes	45	4.5	73.02 (17.63)	0.846	0.87(0.22)	0.964	0.583***
No	959	95.5	72.23 (14.54)		0.92(0.12)		0.533***

*SD* Standard deviation, * < 0.05, ** < 0.01, *** < 0.001

### Ethical consideration

The Clinical Research Ethics Committee of the Chinese University of Hong Kong (CREC–CUHK) approved this study (Ref ID: 2017.093). The respondents who were approached for the survey were required to sign an informed consent form, where the study’s purpose and process and the patients’ rights are clearly indicated.

### Measures

EQ-5D-5L, which was developed by EuroQol, is a generic preference-based tool for measuring HRQoL. It comprised five dimensions, namely, mobility (MO), self-care (SC), usual activities (UA), pain/discomfort (PD) and anxiety/depression (AP); each dimension has five levels (i.e., no problem, slight problems, moderate problems, severe problems, and extreme problems) [21]. EQ-5D-5L, which has 3,125 (5^5^) health states, is the updated version of the previous EQ-5D-3L. With the exception of the descriptive system, EQ-5D also consists of another EQ-VAS part. EQ-VAS records the respondent’s self-rated health on a vertical VAS with two endpoints labelled ‘the best health you can imagine’ and the ‘worst health you can imagine’. This information can also be used as a quantitative measure to reflect the health states of the respondents [3]. In this study, the EQ-5D-5L Traditional Chinese version was utilized [22], and the utility score was estimated using the HK value set [23].

### Statistical analysis

Descriptive statistics were presented to characterize the sample. The mean and standard deviation (SD) of EQ-5D utility and EQ-VAS score were presented. Frequency (n) and proportion (%) were used to describe the background characteristics of the participants and their selection of EQ-5D health states. The bootstrap version of heteroscedastic one-way ANOVA for trimmed means was adopted to compare the mean of the utility index and EQ-VAS on the basis of different population groups [24]. Pearson’s correlation coefficient was used to measure the strength of the linear relationship between EQ-5D utility and EQ-VAS score. The association of EQ-VAS score and EQ-5D profile with other demographic characteristics was assessed using the ordinary least squares linear regression model [25]. In consideration of its heavy ceiling effect (49.6%), the heteroscedastic Tobit model was used to estimate such relationship in utility [12, 26]. With regard to model quality, we examined the log-likelihood using the Akaike information criterion and the Bayesian information criterion [27]. All the analyses were performed using R software (version 4.3.1) [28]. Moreover, missing values were handled listwise. Statistical significance was considered at *p* < 0.05.

## Results

The descriptive statistics and responses to the EQ-5D instrument for the study cohort are provided in Table 1. The mean age of the respondents was 50.91 years, and the respondents were mostly females (52.7%, *n* = 529). The mean utility and EQ-VAS score were 0.92 (SD = 0.13) and 72.27 (SD = 14.69), respectively. Pearson’s correlation coefficient indicated a significantly positive association between EQ-5D utility and EQ-VAS score (*r* = 0.34 ~ 0.71) amongst different demographic groups.

Table 2 presents the most frequently reported EQ-5D health states, the corresponding utility, and the VAS score. In particular, 50.5% of the respondents chose the state of ‘11,111’, followed by the state of ‘11,121’ (16.7%). For VAS, the highest mean score was 72.63 (for the state of ‘11,111’), where the lowest score was 50.0, which corresponded to the utility of 0.496. Table 3 provides the proportion of respondents who reported problems in each level of the five dimensions of EQ-5D. In particular, 98.5% of the respondents reported having no problem with ‘Self-care’, followed by ‘Usual activities’ (96.3%) and ‘Mobility’ (91.5%). Only 58.2% of the respondents reported having some problems with ‘Pain/discomfort’. Moreover, we examined the utility–VAS (U–V) association of each EQ-5D dimension. The overall correlation coefficient was 0.532, and respondents who reported some problems (States ≠ 11,111) exhibited a stronger association (*r* = 0.387 ~ 0.623) than those who reported having no problem (States = 11,111).

**Table 2:** EQ-5D health states, corresponding utility and EQ-VAS

	**State**	**n**	**%**	**Utility score**	**EQ-VAS**	
					**Mean**	**SD**
1	11,111	517	50.49	1.000	72.63	18.41
2	11,121	171	16.70	0.924	60.92	19.50
3	11,122	58	5.66	0.844	62.00	17.51
4	11,131	58	5.66	0.853	65.42	20.83
5	11,112	51	4.98	0.920	63.64	21.11
6	11,132	18	1.76	0.773	53.33	14.72
7	21,121	15	1.46	0.815	71.43	14.35
8	21,131	11	1.07	0.743	60.00	15.55
9	11,133	10	0.98	0.713	50.63	19.54
10	21,111	6	0.59	0.891	78.00	11.51
11	11,113	5	0.49	0.860	70.00	12.75
12	11,141	5	0.49	0.693	55.00	22.91
13	21,122	5	0.49	0.735	65.00	21.21
14	31,132	5	0.49	0.590	52.00	19.56
15	11,123	4	0.39	0.784	51.67	12.58
16	21,132	4	0.39	0.663	50.00	10.00
17	11,222	3	0.29	0.777	63.33	15.28
18	21,123	3	0.29	0.675	63.33	20.82
19	31,131	3	0.29	0.670	50.00	22.91
20	31,231	3	0.29	0.603	55.00	7.07
21	31,332	3	0.29	0.496	50.00	10.00

There are in total of 62 health states, the first 21 represent the 93.5% of participants’ choice

**Table 3:** Distribution within the EQ-5D dimensions and correlation between EQ-5D utility and EQ-VAS

Level	1	2	3	4	5	Health Status	Correlation coefficient
Mobility	919 (91.5%)	57 (5.7%)	25 (2.5%)	3 (0.3%)	0	Status = 11,111	0.495***
						Status ≠ 11,111	0.539***
Self-care	989 (98.5%)	11 (1.1%)	4 (0.4%)	0	0	Status = 11,111	0.513***
						Status ≠ 11,111	0.573*
Usual activities	967 (96.3%)	25 (2.5%)	10 (1.0%)	0	25 (2.5%)	Status = 11,111	0.494***
						Status ≠ 11,111	0.623***
Pain/discomfort	584 (58.2%)	274 (27.3%)	132 (13.1%)	12 (1.2%)	2 (0.2%)	Status = 11,111	0.211***
						Status ≠ 11,111	0.493***
Anxiety/depression	802 (79.9%)	161 (16.0%)	32 (3.2%)	8 (0.8%)	1 (0.1%)	Status = 11,111	0.387***
						Status ≠ 11,111	0.494***
						Overall	0.532***

^*^ *P* < 0.05, ** *p* < 0.01. ***p < 0.001; Status 11,111 = Utility 1.0 (full health)

**Fig. 1:**
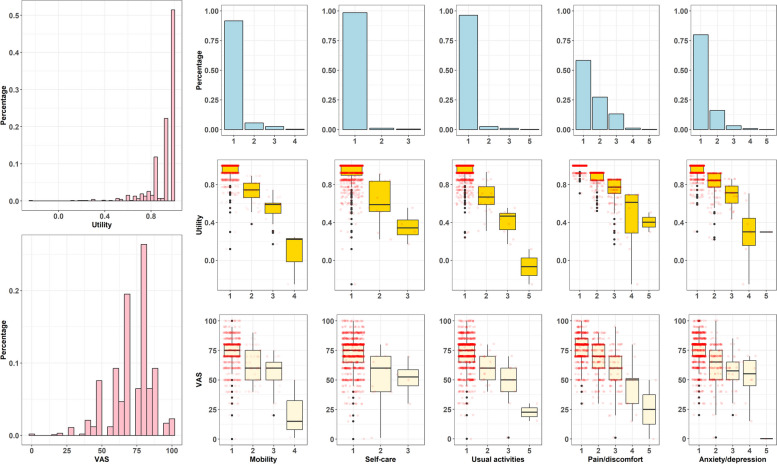
The distribution of EQ-5D utility and EQ-VAS score on each dimension of EQ-5D

Figure 1 shows the distribution of EQ-5D utility and EQ-VAS score in the population sample. The distribution of utility presented a strong negative skew, whereas the distribution of EQ-VAS score was nearly normal. When examining the distribution of each dimension, the pattern was similar to the overall pattern. For EQ-VAS score, however, the distributions were more centralised.

**Fig. 2:**
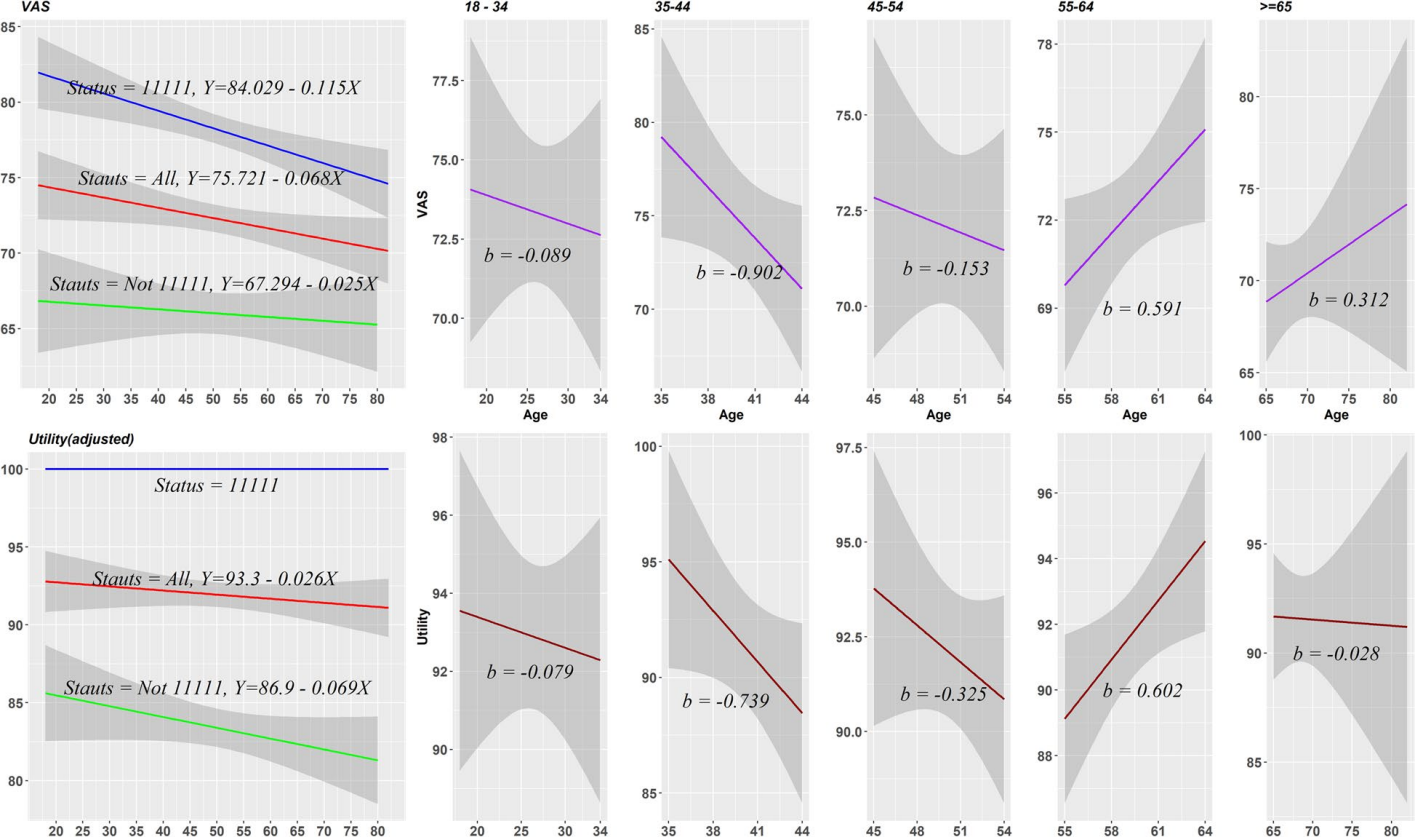
The variation of EQ-5D utility and EQ-VAS score regarding age group

Figure 2 illustrates the variation of utility and EQ-VAS score across different age groups. Overall, older respondents were more likely to report poor health status. However, the decreased rate for EQ-VAS score was more rapid than that for utility [beta (VAS) = 0.068, beta (utility) = 0.026]. Moreover, the curves for age-related variation in health status fluctuated rapidly for utility and EQ-VAS amongst respondents older than 55 years.

Table 3 indicated the only 58.2% or respondents reported having no problem on dimension of ‘Pain/discomfort’ of EQ-5D, followed by ‘Anxiety/depression’ (79.9%). The correlation between utility and EQ-VAS were stronger when respondents reported having some problems for overall (r = 0.532 for not full health, r = 0.494 for full health) and each dimension respectively.

Table 4 further illustrates the relationship amongst utility, EQ-VAS and demographic characteristics. Model 1 shows the EQ-VAS score predicted by the respondents’ response to the EQ-5D profile. The results exhibited a significant relationship between EQ-VAS score and most levels of ‘Mobility’, ‘Pain/discomfort’ and ‘Anxiety/depression’. After adjusting differences in patient characteristics, the EQ-VAS model (Model 2) indicated that government allowance receivers obtained a lower EQ-VAS score, whereas married respondents reported good HRQoL (beta = 4.829, 95% CI 2.021 ~ 7.637). For the utility model (Model 3), females, government allowance receivers, respondents with caregiver and unemployed respondents obtained a remarkably low utility.

**Table 4:** The regression model of EQ-5D utility and EQ-VAS score

	Model 1		Model 2		Model 3	
	Coefficient	95% C.I	Coefficient	95% C.I	Coefficient	95% C.I
Mobility 2	-2.109	(-5.624,1.405)				
Mobility 3	-4.947**	(-10.799,-0.904)				
Mobility 4	-19.063**	(-35.275,-2.851)				
Self-care 2	-9.737	(-17.717,-1.757)				
Self-care 3	-1.764	(-16.071,12.542)				
Usual activities 2	-0.706	(-6.112,4.7)				
Usual activities 3	-3.178	(-13.107,6.751)				
Usual activities 5	-20.767	(-43.086,1.553)				
Pain/discomfort 2	-5.941***	(-7.765,-4.117)				
Pain/discomfort 3	-13.028**	(-15.619,-10.437)				
Pain/discomfort 4	-23.218**	(-31.143,-15.293)				
Pain/discomfort 5	-16.872	(-41.223,7.479)				
Anxiety/depression 2	-7.972**	(-10.13,-5.813)				
Anxiety/depression 3	-11.259***	(-15.817,-6.7)				
Anxiety/depression 4	-2.114	(-12.454,8.225)				
Anxiety/depression 5	-61.259***	(-95.379,-27.138)				
Female			1.406	(-0.651,3.463)	-0.056***	(-0.089,-0.023)
Age			-0.073	(-0.179,0.033)	-0.002	(-0.001,0.004)
Middle school			0.476	(-2.533,3.486)	0.016	(-0.032,0.064)
Senior school			1.964	(-1.058,4.985)	0.037	(-0.011,0.086)
Tertiary or above			2.434	(-1.051,5.92)	0.062**	(0.006,0.118)
Receive allowance			-3.522***	(-6.893,-0.151)	-0.113***	(-0.165,-0.061)
Unemployment			-2.623	(-8.192,2.945)	-0.090***	(-0.177,-0.004)
Full-time student			0.351	(-5.73,6.433)	-0.007	(-0.106,0.092)
Housewife			-2.473	(-5.815,0.87)	-0.048	(-0.101,0.005)
Full-time employment			0.231	(-2.626,3.088)	-0.028	(-0.075,0.019)
Part-time employment			3.795	(-3.184,10.774)	-0.085	(-0.195,0.025)
Live with family			-1.846	(-5.799,2.108)	0.009	(-0.055,0.072)
Live with others			12.74	(-4.608,30.088)	0.301	(-0.003,0.604)
Married			4.829***	(2.021,7.637)	0.032	(-0.013,0.077)
Divorced			1.918	(-3.666,7.501)	0.011	(-0.077,0.1)
Widow			5.969	(-0.471,12.409)	0.024	(-0.077,0.124)
No chronic disease			1.927	(-0.364,4.218)	0.005	(-0.032,0.042)
Have caregiver			0.335	(-4.151,4.821)	-0.082***	(-0.153,-0.011)
AIC	7890.885		8222.814		579.114	
BIC	7979.297		8320.989		677.289	
Adjusted R^2^	0.31		0.32		0.29	

^*^ *p* < 0.05, **p < 0.01, ***p < 0.001; Model 1 is OLS regression model; Model 2 is OLS regression model; Model 3 is Tobit regression model

## Discussion

This study addresses the gap in reporting the association between EQ-5D utility and EQ-VAS score in a primary healthcare setting, which has not been widely reported or discussed elsewhere in Chinese population. From the analysis of the relationship between EQ-5D utility and EQ-VAS score, three key findings were extracted. Firstly, EQ-VAS score is more sensitive than utility in detecting minor variations in age-related health status. Secondly, a consistent and systematic relationship was found between EQ-5D utility and EQ-VAS score. Lastly, EQ-VAS score can provide important information regarding people's health status based on conceptual constructs other than utility.

Our study provides empirical evidence in primary care setting that using EQ-VAS with a single score might be easier for respondents to report their health status compared to using the EQ-5D descriptive system. This finding is consistent with a UK study, where 85% of National Health Service patients completed the EQ-VAS unambiguously [29]. Klimek also noted that the main benefit of using EQ-VAS from a subjective point of view is its ease of use and involved in decision-making process [30]. However, we also found that the EQ-VAS score was highly variable, even for the same health state reported by the EQ-5D descriptive system. These variations between individuals may be due to differences in socioeconomic, physical, or mental factors. Brazier reported that VAS techniques tend to generate meaningless results [4]. Feng, et al. also indicated that caution should be exercised when reporting the results of EQ-VAS, as it may lead to some unusable responses [5]. Nonetheless, in our study, most respondents (90%) clearly defined their health state on the EQ-VAS scale. A significantly positive utility–VAS correlation showed that people understood the meaning of the VAS scale and made reasonable choices based on their understanding. We acknowledge that expectations of HRQoL are closely tied to individuals' subjective experiences. Therefore, we anticipate the development of customized and localized guidelines for the EQ-VAS system, specifically tailored to different subpopulations. This will help mitigate the structural impact on HRQoL evaluation.

We discovered that the equilibrium point between utility and EQ-VAS score is continually shifting towards patients who are experiencing increasing physical or mental discomfort, which supported the argument of Carr et al.’s that experiences constantly shape individual’s health expectations [31]. This finding provides evidence that EQ-VAS and EQ-5D utility score have a dynamic construct for assessing HRQoL. The EQ-VAS seems to be more appropriate and flexible for measuring changes in HRQoL when respondents indicate that they have no health issues, as this is inherent in its design. However, when it comes to utility scores, even if respondents report no problems, a perfect health status is limited to 1.0 [5]. This limitation restricts its ability to accurately reflect changes in health status [32]. Robinson et al. also noted that people's health status is often influenced by contextual factors beyond the health intervention itself [33]. The construct of the EQ-VAS may determine its sensitivity in detecting even minor influences on HRQoL variations.

Previous research on EQ-5D has shown that older individuals are more likely to report a poor HRQoL [27, 34–36]. However, few studies have examined how the association between utility and EQ-VAS affects the magnitude of HRQoL variation across age groups. It is important to report and analyze substantial heterogeneity across different age groups before making any HTA decision. In this study, we observed that both utility and EQ-VAS scores decreased with increasing age. However, the EQ-VAS score decreased more rapidly than the utility score. Nevertheless, when we examined the trajectory of people's reported problems with EQ-5D (health state ≠ 11,111), we found some changes in the results. EQ-VAS score decreased more slowly than utility, which suggests that people with health problems tend to be more conservative, intentionally or unintentionally, in using the VAS scale to report their health status. Previous studies have shown culture [37], response shift [38], focusing illusion [39], and adaptation [31] could potentially explain these findings. This is because the EQ-VAS and utility are based on different conceptual frameworks [29]. However, the degree and direction of the effect that these frameworks have on quantifying people's variation in HRQoL in response to changing health conditions are not well understood.

Age, as another underexplored source of variation that consists of heterogeneity in reporting a population's health status, was investigated in this study. The EQ-5D utility and EQ-VAS score reflected considerable variations in self-reported health state across age groups. These findings are inconsistent with those of previous studies that emphasized the relationship between age and HRQoL, where age was a risk factor for harming HRQoL. Only Quintein reported finding as similar as ours that HRQoL exhibited poor physical HRQoL with increasing age but good outcome for social functioning among cancer patients [40]. Currently, cost-effectiveness analysis (CEA) often relies on a method that uses average preferences from a sample population to represent the preferences of the entire society [41]. However, this approach can be risky as factors like age can introduce biases in estimating the benefits of CEA [42]. This study provides empirical evidence that the influence of age on HRQoL must be considered in preference estimation, and the use of age-specific average preferences may be an optimal method for identifying and adopting heterogeneity and reporting a reasonable arrangement of scarce social care resources. However, considering the absence of evidence-based guidelines to justify which EQ-5D tool is more sensitive to detecting age heterogeneity when reporting HRQoL in different cases, our findings illustrate that reporting the results of utility and EQ-VAS score and providing an unambiguous comparison may be the best choice, particularly for the elderly.

This study demonstrated that even individuals who reported a ‘full health’ (utility = 1.0) status using the EQ-5D descriptive system had an extremely sharp decline in EQ-VAS score with increasing age. This result may suggest that EQ-5D utility has limitations in reflecting people’s real health status at the extreme end of the scale. On the other hand, EQ-VAS score appears to be sensitive to capturing the effect of the natural ageing process on HRQoL. Older people, even those with the same health state defined by EQ-5D descriptive system, tend to have poorer overall health status than young people. Cubi-Molla et al. observed that the health of older individuals tends to exhibit great variability, and the interpretation of the same underlying health state should differ depending on the individual's age [43]. Another study in Netherland emphasized the importance of considering the differences in health valuations between younger and older individuals when selecting or establishing outcome measures [44]. Our study suggests that although the effect of age may be small or uncertain, it should not be disregarded when assessing HRQoL burden changes. Therefore, the use of EQ-VAS is encouraged to estimate magnitude and size differences amongst different age groups in HRQoL.

Furthermore, in this study, the EQ-5D profile accurately predicted the EQ-VAS score. We observed that the regression coefficients for most dimension levels of the EQ-5D descriptive system were appropriately ordered. However, the dimensions of 'Usual activities' and 'Self-care' did not perfectly reflect the variation of the EQ-VAS score. This result differs slightly from the findings of a previous UK study, which indicated a significant relationship between the EQ-VAS score and all the EQ-5D-3L profiles [5]. No similar analysis has been reported for the EQ-5D-5L, therefore, we cannot determine whether the updated five-level version of EQ-5D makes EQ-VAS less sensitive to capturing the variation of health status, or if EQ-VAS cannot reflect the health status of HK people on these two dimensions.

Although EQ-VAS is an alternative method, it is not a true utility instrument [4]. Respondents are not required to trade off anything for a health status, and the value is not calculated based on preference. Therefore, we suggest that the EQ-VAS might be useful in the clinical practice for comparing the effectiveness of clinical interventions. However, for political decision-making, utility may be more appropriate for conducting HTA to allocate health and social resources [45]. The debate over which technique or variant is more appropriate for measuring and valuing health continues [4]. Current research is the first and an important step in exploring the meaning and mechanism of the EQ-VAS for populations with different socioeconomic characteristics. Researchers should exercise caution when reporting EQ-5D results, as the disparity between the utility and EQ-VAS data could potentially suggest methodological issues in data collection or analysis.

Although the discussion of this study mainly focuses on the methodology, the findings also have practical implications. Our findings support that EQ-VAS is a valid and simple instrument for rapidly assessing an individual's perceived health status. It can be widely used in primary care settings to assess the effectiveness of clinical interventions. Its simplicity and flexibility can be valuable in supporting doctor-patient communication and improving patient-centered care in HK. The performance of EQ-VAS in different patient groups should be further explored.

Several limitations of this study should be addressed. The primary limitation is that all participants were recruited from one GOPC, Although the difference of most key background characteristics between our sample and general population was statistically insignificant, selection bias might be existed. Given potential variations in patients' demographics, comorbidities, laboratory data, and medication usage across GOPCs, there may be a limited understanding of the Hong Kong population and insufficient support for robust statistical analysis. Future studies should consider collecting data from different local healthcare services. multiple GOPCs. By including a diverse range of GOPCs, we can obtain a more representative sample and enhance the overall validity and reliability of our results. Additionally, this was a cross-sectional design, thus, no causal relationship can be developed. Third, all participants in this study were primary care patients. Compared to the general population, our sample was slightly older, the comparisons between EQ-5D-5L utility score and EQ-VAS in local general population are unknown and need further analysis. Last, in this study, due to self-reported, no clinical data was collected by participants. The comparisons between EQ-5D utility score and EQ-VAS regarding different clinical conditions are not presented.

## Conclusion

This study compares the EQ-5D utility score and EQ-VAS in a primary healthcare setting in HK. Positive correlations were observed between the utility score and EQ-VAS, indicating their reliability in estimating HRQoL. When individuals reported being in full health, EQ-VAS was more sensitive than the utility score in detecting minor variations in health status. However, when individuals reported having some health problems, the descriptive system was more effective. Additionally, the study found that age has a significant impact on HRQoL. Further research is encouraged to assess HRQoL profiles over time and capture informative variations.

### Supplementary Information

**Additional file 1:** The comparison between the sample and general population in HK. **Table A1.** The demographics of participant from phase three survey (*N*=1004).

## Data Availability

The datasets generated during and/or analyzed during the current study are available from the corresponding author on reasonable request.
