# Supplementary material for: Health-related quality of life in primary care patients: a comparison between EQ-5D-5L utility score and EQ-visual analogue scale
Source: Health Qual Life Outcomes. 2024 Jan 3;22:2. doi: 10.1186/s12955-023-02215-w (PMC10765691; doi:10.1186/s12955-023-02215-w)
Supplement: Supplementary file 1 — Additional file 1: The comparison between the sample and general population in HK. Table A1. The demographics of participant from phase three survey (N=1004). [file 12955_2023_2215_MOESM1_ESM.docx]

Table A1 The demographics of participant from phase three survey (N=1004)

|  | Participant | | General population |  |  |
| --- | --- | --- | --- | --- | --- |
|  | n | % | % |  |  |
| Gender |  |  |  |  |  |
| Male | 475 | 47.3 | 46.1 |  | 0.89 |
| Female | 529 | 52.7 | 53.9 |  |  |
| Age group (18-80) |  |  |  |  |  |
| 18-34 | 175 | 17.4 | 37.4 |  | 0.03 |
| 35-44 | 113 | 11.3 | 15.6 |  |  |
| 45-54 | 218 | 21.7 | 16.8 |  |  |
| 55-64 | 325 | 32.4 | 14.8 |  |  |
| >65 | 155 | 15.4 | 15.3 |  |  |
| NA | 18 | 1.8 |  |  |  |
| Education |  |  |  |  | 0.47 |
| No education/ Primary | 151 | 15.0 | 18.9  50.3 |  |  |
| Secondary | 259 | 25.8 |  |  |  |
| Post-secondary | 334 | 33.3 |  |  |  |
| Tertiary or above | 260 | 25.9 | 30.8 |  |  |
| Receive government allowance |  |  |  |  | 0.81 |
| Yes | 93 | 9.3 | 11.1 |  |  |
| No | 911 | 90.7 | 88.9 |  |  |
| Current living status |  |  |  |  |  |
| Live alone | 71 | 7.1 |  |  |  |
| Live with family/ others | 927 | 92.3 |  |  |  |
| Live in institution2 | 3 | 0.3 |  |  |  |
| NA | 3 | 0.3 |  |  | 0.46 |
| Marriage status |  |  |  |  |  |
| Single | 233 | 23.2 | 30.1 |  |  |
| Married | 712 | 70.9 | 58.4 |  |  |
| Divorce | 34 | 3.4 | 4.6 |  |  |
| Widow | 25 | 2.5 | 6.4 |  |  |
| Current working status |  |  |  |  |  |
| Retired | 232 | 23.10 |  |  |  |
| Unemployment | 36 | 3.60 |  |  |  |
| Full-time student | 39 | 3.90 |  |  |  |
| Housewife | 189 | 18.80 |  |  |  |
| Full-time employment | 489 | 48.70 |  |  |  |
| Part-time employment | 19 | 1.90 |  |  |  |
| Having chronic condition# |  |  |  |  |  |
| Yes | 250 | 24.9 |  |  |  |
| No | 754 | 75.1 |  |  |  |

# 6 people reported uncertain
